# Thorough QT Study on the Effect of Therapeutic and Supratherapeutic Dosing of Givinostat in Healthy Volunteers

**DOI:** 10.1002/cpdd.70047

**Published:** 2026-03-22

**Authors:** Eugenio Mercuri, Barry Byrne, Tracey Willis, John Bourke, Paolo Bettica, Sara Cazzaniga, Hongqi Xue, Borje Darpo

**Affiliations:** ^1^ Pediatric Neurology Institute Catholic University and Nemo Pediatrico Fondazione Policlinico Gemelli IRCCS Rome Italy; ^2^ Institute for Child Health Policy University of Florida Gainesville Florida USA; ^3^ The Robert Jones and Agnes Hunt Orthopaedic Hospital NHS Foundation Trust Oswestry UK; ^4^ The John Walton Muscular Dystrophy Research Centre, Freeman Hospital Newcastle University and Newcastle Hospitals NHS Foundation Trust Newcastle upon Tyne UK; ^5^ Italfarmaco S.p.A. Milan Italy; ^6^ Clario, Philadelphia Pennsylvania USA

**Keywords:** electrocardiogram, givinostat, histone deacetylase inhibitor, pharmacokinetic, QTcF

## Abstract

Givinostat is a class I/II histone deacetylase inhibitor indicated for Duchenne muscular dystrophy (DMD). The study evaluated the effect of therapeutic and supratherapeutic givinostat doses on the QT/QTc interval. Healthy volunteers received each treatment—givinostat hydrochloride monohydrate oral suspension as a therapeutic (100 mg) or supratherapeutic (300 mg) dose, placebo oral suspension, or moxifloxacin oral tablet (positive control, 400 mg)—according to a block randomization scheme. Cardiodynamic assessments were paired with pharmacokinetic samples. A small, clinically non‐relevant effect on mean placebo‐corrected, change‐from‐baseline QTcF (∆∆QTcF) of 5.5 ms was seen after givinostat 100‐mg dose. Clinically relevant QTc prolongation was observed with the supratherapeutic dose, with a mild ∆∆QTcF increase of 13.6 ms. A delay of ≈3 h between T_max_ and the largest effect on the QTc interval was seen for both doses. In the concentration‐QTc analysis, an E_max_ model captured the data better than the prespecified linear model and showed that an effect on ∆∆QTcF exceeding 10 ms could be excluded within the full range of observed givinostat concentrations in this study and up to ≈745 ng/mL. Givinostat at the maximum labeled dose (up to 53.2 mg twice daily for DMD) is not expected to pose a QT prolongation risk.

Givinostat is a class I and II histone deacetylase (HDAC) inhibitor^1^ that is indicated for Duchenne muscular dystrophy (DMD) treatment in patients aged 6 years and older,^2^, ^3^, ^4^, ^5^ based on the efficacy and safety findings from the Epigenetic Rescue of Dystrophin Dysfunction (EPIDYS) study, a double‐blind, randomized, placebo‐controlled phase 3 trial (NCT02851797).^6^ This novel nonsteroidal drug has also been evaluated in various diseases, including polycythaemia vera,^7^ systemic‐onset juvenile idiopathic arthritis,^8^ and Becker muscular dystrophy.^9^

Historically, clinically significant electrocardiogram (ECG) changes, including QT prolongation, have been observed in some clinical studies with HDAC inhibitors.^10^, ^11^ Givinostat was designed to overcome the safety and tolerability challenges observed with earlier compounds in this class. HDAC inhibition by givinostat leads to increased histone acetylation, resulting in a more open and relaxed chromatin structure and enhanced transcription of genes involved in muscle repair.^12^ Unlike other HDAC inhibitors, givinostat has shown efficacy at dose levels that are generally well tolerated.^6^, ^12^

This study evaluated the effect of givinostat on cardiac repolarization via a thorough QT/corrected QT (QTc) study in healthy volunteers.^13^, ^14^ The primary objective was to evaluate the impact of therapeutic and supratherapeutic doses of givinostat on the QT/QTc interval. The secondary objectives were to evaluate additional ECG measures (e.g., heart rate [HR]), pharmacokinetic (PK) parameters, and the safety and tolerability of therapeutic and supratherapeutic doses of givinostat.

## Methods

This study was a single‐center, randomized, partially double‐blinded, single‐dose, placebo‐ and positive‐controlled, four‐way crossover study (NCT04821063) in healthy adult participants. The study was aligned with the International Council for Harmonisation E14 guidelines for clinical evaluation of QT interval prolongation for non‐antiarrhythmic drugs and was also reviewed by the US Food and Drug Administration (FDA) Interdisciplinary Review Team for QT studies.^13^, ^14^, ^15^ It was approved by an independent ethics committee (Advarra; Aurora, Ontario, Canada), was conducted in accordance with Good Clinical Practice, and was in compliance with the Declaration of Helsinki. The trial was conducted at Syneos Health Clinique Inc, Québec (Québec), Canada. All participants provided written informed consent to participate in the study.

### Study Population

Eligible participants were male or female nonsmokers between the ages of 18 and 55 years in good health based on the absence of a clinically significant history of disease (e.g., cardiovascular and metabolic conditions), physical examination, routine laboratory tests (e.g., blood chemistry and urinalysis), vital signs, and 12‐lead ECG. Nonsmoking status was confirmed by urine cotinine test at screening or at baseline day ‐1 (prior to dosing). Participants were excluded if there were cardiac arrhythmias, positive urine drug screen, positive pregnancy test, or exposure to givinostat for a medical condition or previous clinical trial (within 30 days prior to dosing). A complete list of inclusion and exclusion criteria can be found in the Supporting Information.

### Study Design and Treatments

On day ‐1, participants were randomized to each treatment according to a 4‐period, 12‐sequence randomization scheme; periods were separated by a washout of 7 days. Participants underwent a screening visit to determine trial eligibility within 28 days before study drug administration. During each period, participants fasted for ≥10 h overnight and were observed in the clinic for ≥12 h before dosing. On the morning of day 1 in each period, participants were administered the following under fasted conditions:

- Therapeutic dose of givinostat (100 mg givinostat hydrochloride monohydrate) administered as 1 × 10 mL oral suspension (10 mg/mL) plus 2 × 10 mL of matching placebo oral suspension (30 mL total).
- Supratherapeutic dose of givinostat (300 mg givinostat hydrochloride monohydrate) administered as 3 × 10 mL oral suspension (10 mg/mL).
- Placebo, administered as 3 × 10 mL matching oral suspension.
- Moxifloxacin 400‐mg tablet, used as a positive control.^16^

Oral suspension of 10 mg/mL givinostat hydrochloride monohydrate equates to 8.86 mg/mL of givinostat‐free base in the oral suspension. For the first 4 h after study drug administration, food was not allowed, and participants were requested to remain in a supine or semi‐reclined position and to avoid sleeping. Clinical trials have evaluated single and multiple givinostat doses ranging between 20 and 100 mg twice a day and up to 600 mg (single dose).^6^, ^7^, ^17^, ^18^, ^19^, ^20^ The supratherapeutic dose (300 mg) was selected since 400‐ and 600‐mg doses have already been administered and demonstrated tolerability in early studies of givinostat in healthy, young participants.^19^ Population PK (PPK) analyses have shown that the PK profiles and parameters were similar between healthy participants and patients.^21^

Moxifloxacin is a well‐characterized positive control in thorough QT/QTc studies. A single 400‐mg dose of moxifloxacin has been shown to produce QTc prolongation between 8 and 16 ms, with peak concentrations occurring between 1 and 4 h.^22^, ^23^, ^24^ Moxifloxacin was administered as a commercially available tablet in accordance with its established use as a positive control for assay sensitivity in thorough QT/QTc studies. Although formulation‐dependent differences in absorption rate may occur, these do not affect the assessment of assay sensitivity or the interpretation of givinostat concentration‐QTc relationships.

### Randomization and Blinding

The randomization schedule was generated using Statistical Analysis System (SAS), Version 9.4. Givinostat 100 mg and 300 mg and placebo were prepared individually by an unblinded team at the site pharmacy, according to the randomization schedule, and were administered in a double‐blinded fashion by the site team; moxifloxacin was administered as open‐label treatment. The ECG analyst was blinded to treatment, study period, time point of ECG recording, and participant details (e.g., laboratory results).

### Primary and Secondary Endpoints

The primary endpoint was the placebo‐corrected change from baseline in QTcF (Fridericia's correction; ΔΔQTcF). Secondary endpoints included change from baseline in QTcF (ΔQTcF), PR interval (ΔPR), QRS interval (ΔQRS), and HR (ΔHR); placebo‐corrected ΔPR (ΔΔPR), ΔQRS (ΔΔQRS), and ΔHR (ΔΔHR); categorical outliers for QTcF, PR, QRS, and HR; and frequency of treatment‐emergent changes of T‐wave morphology and U‐wave presence. PK endpoints included plasma and urine parameters for givinostat and its metabolites and plasma parameters for moxifloxacin. Safety and tolerability of givinostat were evaluated through assessments of adverse events (AEs), vital signs, ECGs, and clinical laboratory parameters.

### Cardiodynamic Evaluations

All ECG data were collected using a Global Instrumentation (Manlius, NY, USA) M12R ECG continuous 12‐lead digital recorder. The cardiodynamic assessment was performed through 12‐lead ECGs, and continuous ECG recordings were performed from 1 h prior to dosing in each treatment period until 36 h postdose. Up to 10 replicate ECGs were extracted at −45, −30, and −15 min predose and 0.5, 1, 1.5, 2, 2.5, 3, 3.5, 4, 5, 6, 7, 8, 12, 24, and 36 h postdose, using the EPQT method.^22^ Participants rested in a supine position for ≥10 min before and 5 min after each ECG extraction, whenever possible. Treatment‐emergent changes for T‐wave morphology and U‐wave presence were assessed and recorded. For all continuous ECG parameters in each period, baseline was defined as the average of the measured ECG intervals from the 3 ECG time points (−45, −30, and −15 min) prior to treatment administration on day 1. The QT and RR interval for each beat was used for HR correction. The QTcF was defined as QTcF (ms) = QT (ms)/[RR(ms)/1000]^1/3^.

### Bioanalytical Assay Methodologies for Givinostat and Moxifloxacin

Plasma concentrations of givinostat and moxifloxacin were quantified using validated liquid chromatography with tandem mass spectrometry (LC‐MS/MS) methods. The bioanalytical method for givinostat determination involved automated liquid–liquid extraction with methyl tert‐butyl ether and LC‐MS/MS analysis using the structural analogue ITF2400 as the internal standard. The limit of quantification was 1.00 ng/mL for givinostat. The intra‐day accuracy and precision were characterized by a percentage of bias of −8.33% to 6.16% and a coefficient of variation of 0.92 to 12.77, and the inter‐day accuracy and precision were characterized by a percentage of bias of −2.00% to 2.41% and a coefficient of variation of 3.71 to 6.12. The stability of givinostat in human plasma was verified for 203 days at −80°C with four freeze and thaw cycles.

The bioanalytical method for moxifloxacin determination involved solid phase extraction and LC‐MS/MS analysis using the structural analogue gatifloxacin as the internal standard. The limit of quantification was 10.02 ng/mL for moxifloxacin. The intra‐day accuracy and precision were characterized by a percentage of bias of −9.13% to 10.50% and a coefficient of variation of 0.49 to 5.92, and the inter‐day accuracy and precision were characterized by a percentage of bias of −3.84% to 4.30% and a coefficient of variation of 3.78 to 5.54. The stability of moxifloxacin in human plasma was verified for 1165 days at −20°C with four freeze and thaw cycles.

### Pharmacokinetic Assessments

Blood samples were collected after the ECG assessment, with a total of 19 blood samples obtained per period for PK analyses at the following time points: predose and at 0.5, 1, 1.5, 2, 2.5, 3, 3.5, 4, 5, 6, 7, 8, 12, 24, 36, 48, 60, and 72 h postdose. Urine samples for PK analysis were collected predose (within 2 h before dosing) and one time during the following intervals: 0 to 8 h, 8 to 24 h, 24 to 48 h, and 48 to 72 h postdose. Plasma concentrations for givinostat and its metabolites and moxifloxacin were used to calculate PK parameters by standard noncompartmental methods, including area under the concentration–time curve (AUC) from time zero to time of the last nonzero concentration (AUC)_0‐t_; AUC from time zero to 12 h (AUC_0‐12_); AUC from time zero to infinity (extrapolated) (AUC_0‐inf_); maximum observed plasma concentration (C_max_), residual area; time of observed C_max_ (T_max_); elimination half‐life calculated as ln (2)/K_el_ (t_1/2 el_); elimination rate constant (K_el_); apparent total body clearance (Cl/F), calculated as dose/AUC_0‐inf_; and apparent volume of distribution (V_d_/F), calculated as dose/K_el_ × AUC_0‐inf_. Urine PK parameters included cumulative urinary excretion from time zero to time t (Ae_0‐t_), maximum rate of urinary excretion (R_max_), time of maximum rate of urinary excretion (T_Rmax_), and renal clearance (Clr). PK analyses were performed using Phoenix WinNonlin version 8.0, which was validated by Syneos Health.

### Statistics

#### Concentration‐QTc Analysis

The concentration‐QTc analysis method is modeled from a previous study by the International Consortium for Innovation and Quality in Pharmaceutical Development and the Cardiac Safety Research Consortium (IQ‐CSRC).^25^ The relationship between plasma concentrations of givinostat and ΔQTcF was quantified using a linear mixed‐effects modelling approach. Pooled data from the therapeutic dose of givinostat (100 mg) and supratherapeutic dose of givinostat (300 mg), as well as placebo data, were included in the same model. The model included ΔQTcF as the dependent variable, givinostat plasma concentrations as the explanatory variate (0 for placebo), centered baseline QTcF (i.e., baseline QTcF for individual participant minus the population mean baseline QTcF for all participants within the same treatment period) as an additional covariate, treatment (active = 1 or placebo = 0) and time as fixed effects, and random effects on the intercept and slope per participant.^26^ From the model, the slope and the treatment effect‐specific intercept (defined as the difference between active and placebo) were estimated together with the two‐sided 90% CI. The geometric mean of the individual C_max_ values for participants in the 100‐ and 300‐mg dose groups was determined separately. The predicted effect and its two‐sided 90% CI for ΔΔQTcF (i.e., slope estimate × concentration + treatment effect‐specific intercept) at this geometric mean C_max_ were obtained. If the upper bound of the two‐sided 90% CI of the predicted QTc effect (ΔΔQTcF) was below 10 ms at clinically relevant plasma levels, it can be concluded that givinostat did not cause clinically relevant QTc interval prolongation within the observed plasma concentration ranges.

Additional exploratory analyses (via graphical displays and/or model fitting) were used for the assessment of model assumptions, including no drug effect on HR (Assumption 1), the selected primary QT correction method should be independent of HR (Assumption 2), no time delay (hysteresis) between drug concentration and ΔΔQTcF (Assumption 3), and linear concentration‐QTc relationship (Assumption 4).^26^ The choice of the pharmacodynamic model (linear vs. nonlinear) was also justified. For Assumption 3, hysteresis was assessed based on joint graphical displays of the least‐squares (LS) mean ΔΔQTcF for each postdose time point from the by‐time point analysis and the mean concentrations of givinostat connected in temporal order by dose. To assess assay sensitivity, a linear mixed‐effects model similar to that used for the primary analysis was used to evaluate the relationship between moxifloxacin plasma concentration and ΔQTcF.

#### By‐Time Point Analysis

The by‐time point analysis for QTcF was based on a linear mixed‐effects model with ΔQTcF as the dependent variable; period, sequence, time, treatment (therapeutic dose of givinostat, supratherapeutic dose of givinostat, and placebo), and time‐by‐treatment interaction as fixed effects; and baseline QTcF as a covariate. An unstructured covariance matrix was specified for the repeated measures at postdose time points for participants within the treatment period. The model also included a participant‐specific random effect. For HR, PR, and QRS interval, the analysis was based on the change‐from‐baseline postdosing values (ΔHR, ΔPR, and ΔQRS), and the same (by‐time point analysis) model was used as described for QTcF.

#### Categorial Analysis

The results for categorical outliers were based on treatment‐emergent events (new findings compared with baseline). T‐wave morphology and presence of U‐waves were summarized in frequency tables with counts and percentages for both the number of participants and the number of time points. A participant or time point was determined as an outlier if the following criteria were met for the ECG intervals: treatment‐emergent QTcF values of >450 and ≤480 ms, >480 and ≤500 ms or >500 ms when not present at baseline; increases from baseline of >30 and ≤60 ms or >60 ms; increase of PR from baseline >25% resulting in PR >200 ms; increase of QRS from baseline >25% resulting in QRS >120 ms; decrease of HR from baseline >25% resulting in HR <50 bpm; increase of HR from baseline >25% resulting in HR >100 bpm.

#### Safety

AEs were recorded and evaluated for their duration, seriousness, severity, and relationship to the study medication. The severity of AEs was assessed and graded according to the most recently published National Cancer Institute Common Terminology Criteria for AE (CTCAE). Serious adverse events (SAE) were any event that met the following criteria: death; life‐threatening, inpatient hospitalization or prolongation of existing hospitalization; persistent or significant disability/incapacity; and congenital anomaly/birth defect in the offspring of a participant. An adverse drug reaction (ADR) was considered for all noxious and unintended responses related to any study dose. A suspected, unexpected, serious adverse reaction (SUSAR) was defined as an ADR that was serious and unexpected. Safety and tolerability data were reported using descriptive statistics. All statistical analyses were performed using the statistical software SAS, Version 9.4.

#### Sample Size Calculation

We estimated that a sample size of 28 evaluable participants would provide >95% power to exclude that givinostat caused a >10‐ms QTc effect at clinically relevant plasma levels, based on the upper bound of the two‐sided 90% CI of the model‐predicted QTc effect at the observed geometric mean C_max_ of givinostat. The sample size calculation assumed a one‐sided 5% significance level, a small underlying effect of givinostat of 3 ms, and an SD of the ΔQTcF of 8 ms for both givinostat and placebo. The sample size for assay sensitivity was also considered. Demonstrating the sensitivity of the concentration‐QTc analysis required that the lower bound of the two‐sided 90% CI of the predicted QTc effect (ΔΔQTcF) of a single dose of 400‐mg moxifloxacin exceed 5 ms. With 28 evaluable participants, a statistical power of ≥95% could be obtained, provided the within‐participant SD of ΔQTcF did not exceed 6.9 ms.

## Results

### Participant Disposition, Demographics, and Baseline Characteristics

A total of 34 participants were enrolled in the study, and 31 participants were dosed. Of these, 29 participants completed all treatment periods (Figure S1, Supporting Information). Two participants were not dosed with givinostat 100 mg in period 2 owing to invalid laboratory results. However, these participants were dosed in period 3 (givinostat 300 mg) and period 4 (moxifloxacin). The majority of participants were White (87.1%) and male (61.3%) (Table 1). The mean age was 40.6 years, and the mean body mass index was 25.7 kg/m^2^.

**Table 1:** Demographics and Baseline Characteristics of the Safety Population

	Givinostat 100 mg, n = 29	Givinostat 300 mg, n = 31	Placebo, n = 31	Moxifloxacin, n = 31	Overall, n = 31
Age (y), mean (SD)	40.7 (10.9)	40.6 (10.8)	40.6 (10.8)	40.6 (10.8)	40.6 (10.8)
Female, n (%)	12 (41.4)	12 (38.7)	12 (38.7)	12 (38.7)	12 (38.7)
Ethnicity					
Hispanic or Latino	9 (31.0)	10 (32.3)	10 (32.3)	10 (32.3)	10 (32.3)
Not Hispanic or Latino	20 (69.0)	21 (67.7)	21 (67.7)	21 (67.7)	21 (67.7)
Race					
Asian	1 (3.4)	1 (3.2)	1 (3.2)	1 (3.2)	1 (3.2)
Black	2 (6.9)	3 (9.7)	3 (9.7)	3 (9.7)	3 (9.7)
White	26 (89.7)	27 (87.1)	27 (87.1)	27 (87.1)	27 (87.1)
BMI (kg/m^2^), mean (SD)	25.5 (2.5)	25.7 (2.4)	25.7 (2.4)	25.7 (2.4)	25.7 (2.4)

BMI, body mass index.

### Cardiodynamic ECG Results

**Figure 1:**
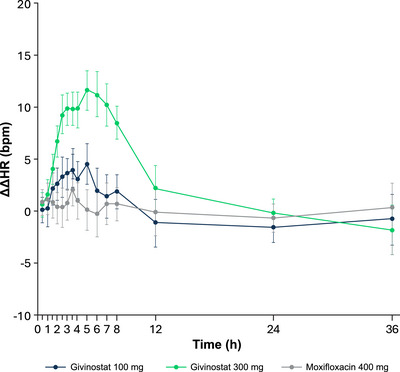
Placebo‐corrected change‐from‐baseline HR (ΔΔHR) across time points (QT/QTc population). LS mean and 90% CI based on a linear mixed‐effects model: ΔHR = time + treatment + time × treatment + baseline HR + period + sequence and a random intercept per participant ID. An unstructured covariance structure was used to specify the repeated measures (postdose time points for participants within the treatment period), and an unstructured covariance structure was used to specify the participant‐specific random effect. bpm, beats per minute; HR, heart rate; LS, least‐squares; QTc, corrected QT.

Baseline ECG parameters across treatment periods were within expectations for healthy adults, with mean HR between 58.4 and 59.7 bpm, mean QTcF between 399.7 and 401.1 ms, mean PR between 144.9 and 148.2 ms, and mean QRS between 106.2 and 106.5 ms. The LS mean ΔΔHR after givinostat 100 mg ranged between 1.4 and 4.5 bpm from 2 to 8 h postdose (Figure 1). From 2.5 to 7 h postdose, the LS mean ΔΔHR after givinostat 300 mg was approximately 10 bpm, with the largest mean effect being 11.6 bpm at 5 h. There were no tachycardic or bradycardic outliers.

**Figure 2:**
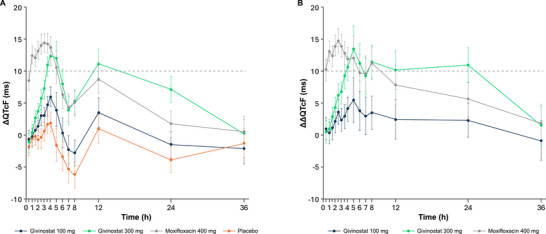
Change‐from‐baseline QTcF (ΔQTcF) (A) and placebo‐corrected change‐from‐baseline QTcF (ΔΔQTcF) (B) across time points (QT/QTc population). LS mean and 90% CI based on a linear mixed‐effects model: ΔQTcF = time + treatment + time × treatment + baseline QTcF + period + sequence and a random intercept per participant ID. An unstructured covariance structure was used to specify the repeated measures (postdose time points for participants within the treatment period), and an unstructured covariance structure was used to specify the participant‐specific random effect. LS, least‐squares; QTc, corrected QT; QTcF, Fridericia's formula correction.

The largest LS mean change‐from‐baseline QTcF (ΔQTcF) was 6.0 ms at 4 h postdose with givinostat 100 mg, whereas the largest mean value was 12.4 ms after givinostat 300 mg (Figure 2). For moxifloxacin 400 mg, the LS mean ΔQTcF increased by 14.5 ms at 3 h postdose. For givinostat 100 mg, the LS mean ΔΔQTcF increased successively from 0.5 h postdose to a peak effect of 5.5 ms at 5 h. For givinostat 300 mg, the LS mean ΔΔQTcF increased to a peak of 13.6 ms at 5 h (Figure 2). Across all postdose time points, the upper bound of the 90% CI of ∆∆QTcF was <10 ms for givinostat 100 mg, indicating no clinically relevant QTc prolongation. After dosing with givinostat 300 mg, the LS mean ΔΔQTcF remained at or slightly above 10 ms until 24 h postdose, after which the effect declined to 1.5 ms at 36 h postdose. After dosing with moxifloxacin 400 mg, an increase of LS mean ΔΔQTcF was observed with a peak value of 14.8 ms (90% CI, 12.84‐16.71) at 2.5 h postdose.

Givinostat 100 and 300 mg did not have a clinically relevant effect on PR and QRS intervals, and there were no PR or QRS outliers. There were no treatment‐emergent T‐wave morphology changes or U‐waves. There were no participants with QTcF >480 ms or ΔQTcF >60 ms.

### Pharmacokinetics Results

The highest mean plasma concentrations of givinostat were observed at 2 h postdose for both the 100‐ and 300‐mg dose (Figure S2, Supporting Information). The mean (SD) C_max_ for givinostat 300 mg was 409.7 (133.7) ng/mL, which was approximately four times greater than the mean (SD) C_max_ for givinostat 100 mg at 102.8 (30.6) ng/mL. The AUCs followed a similar pattern with givinostat 300 mg resulting in values approximately three to four times greater than those observed with givinostat 100 mg (Table S1, Supporting Information). The elimination half‐life for givinostat was 3 h longer for givinostat 300 mg (11 h) compared with givinostat 100 mg (8 h). Twelve participants were randomly selected for urinary and plasma metabolite analysis. Similar ratios for the rate and extent of metabolite formation of the two main metabolites (ITF2374 and ITF2375) were observed. Descriptive results for urine assessment can be found in Table S1, Supporting Information.

### Concentration‐QTc Analysis Results

**Figure 3:**
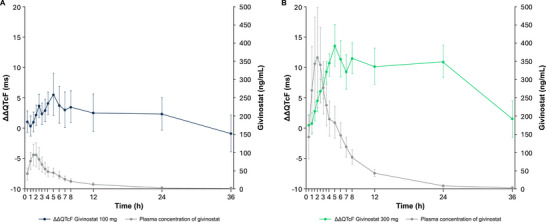
Joint plot of givinostat (A) 100‐mg and (B) 300‐mg plasma concentrations and ΔΔQTcF over time (QT/QTc population, PK/QTc population). Error bars for ΔΔQTcF are 90% CI from by‐time point statistical modeling, and the error bars for concentration are 90% CI from descriptive statistics. PK, pharmacokinetic; QTc, corrected QT; QTcF, Fridericia's formula correction.

A delay of 3 h between peak concentrations of givinostat and the largest QT effects was observed for the 100‐ and 300‐mg doses (Assumption 3). The median T_max_ was approximately 2 h for both doses, whereas the highest QT effects (ΔΔQTcF) were observed at time points with much lower concentrations for both doses, thereby indicating the presence of hysteresis (Figure 3).

**Figure 4:**
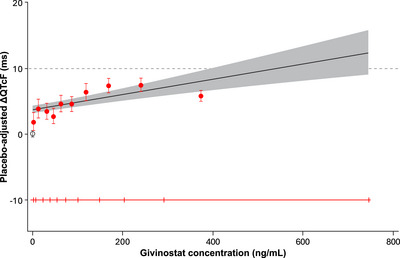
Model‐predicted ΔΔQTcF (mean and 90% CI) and estimated placebo‐adjusted ΔQTcF (mean and 90% CI) across deciles of givinostat plasma concentrations (PK/QTc population). The solid black line with gray shaded area denotes the model‐predicted mean ΔΔQTcF with 90% CI, which is calculated from the equation ΔΔQTcF (ms) = 3.73 (ms) + 0.012 (ms per ng/mL) × givinostat concentration (ng/mL). The red‐filled circles with vertical bars denote the estimated mean placebo‐adjusted ΔQTcF (ΔΔQTcF) with 90% CI displayed at the associated median plasma concentration within each decile for givinostat, among which the individually estimated placebo‐adjusted ΔQTcF*i,k* (ΔΔQTcF*i,k*) equals the individual ΔQTcF*i,k* for participant i administered with givinostat at time point k minus the estimation of time effect at time point k. The black circle with vertical bars denotes the mean placebo‐adjusted ΔQTcF with 90% CI for placebo at a concentration of 0. The horizontal red line with notches shows the range of concentrations divided into deciles for givinostat. The area between each decile represents the point at which 10% of the data are present; the first notch to second notch denotes the first 10% of the data, the second notch to third notch denotes the 10%‐20% of the data, and so on. PK, pharmacokinetic; QTc, corrected QT; QTcF, Fridericia's formula correction.

**Figure 5:**
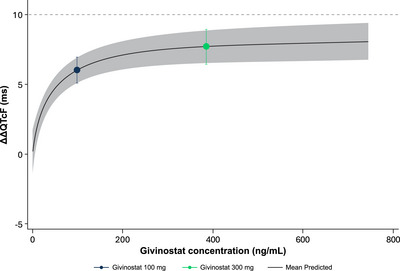
Model‐predicted ΔΔQTcF interval (mean and 90% CI) at geometric mean peak givinostat concentrations for givinostat E_max_ model (PK/QTc population). The solid black line with gray shaded area denotes the model‐predicted mean (90% CI) ΔΔQTcF. The vertical blue and green lines denote the estimated mean (90% CI) ΔΔQTcF with plotted points at the geometric mean C_max_ of givinostat by dose group. The pointwise 90% CI for the mean predicted ΔΔQTcF was derived based on the E_max_ model. E_max_, maximum effect; PK, pharmacokinetic; QTc, corrected QT; QTcF, Fridericia's formula correction.

The goodness‐of‐fit plot showed that the predicted ΔΔQTcF values are close to the estimated placebo‐adjusted ΔQTcF across high plasma concentration levels, except in 1st, 4th, 7th, 8th, and 10th deciles, suggesting that the proposed linear model did not provide an acceptable representation of the relationship between placebo‐adjusted ΔQTcF and givinostat concentration (Figure 4). The linear mixed‐effects model did not provide an optimal fit to the data in the highest (10th) decile of plasma concentration (i.e., concentrations above ∼290 ng/mL) and seemed to overestimate the effect on ΔΔQTcF at high concentrations. The relationship between givinostat plasma concentrations and predicted QTcF was therefore also investigated using an E_max_ model, which captured the data better than the linear mixed‐effects model at higher‐end plasma concentrations (Figure S3, Supporting Information; Table S2, Supporting Information). The details for the E_max_ model are described in the Supporting Information. The Akaike Information Criterion (AIC) value from the E_max_ was smaller than the AIC value from the linear mixed‐effects model (8669.1 vs. 8682.6). The E_max_ was 8.45 ms (90% CI: 6.50 to 10.40), and the estimated EC_50_ was 40.96 ng/mL (90% CI: 14.44 to 67.47). The E_max_ and EC_50_ were statistically significant at the 0.1 significance level. The treatment effect‐specific intercept from the E_max_ model was small (0.078 ms with 90% CI, −1.55 to 1.71) and not statistically significant at the significance level of 0.1, while the treatment effect‐specific intercept from the linear mixed‐effects model was large (3.73 ms) and statistically significant. Based on the E_max_ model, a QT effect (ΔΔQTcF) >10 ms can be excluded within the full range of givinostat plasma concentrations observed in this study and up to ≈745 ng/mL (Figure 5). The linear model with a treatment effect‐specific intercept was used in the assay sensitivity analysis for moxifloxacin. The slope of the relationship was positive and statistically significant (0.0075 ms per ng/mL; 90% CI, 0.00629‐0.00881), and the lower bound of the two‐sided CI of the predicted QT effect at the geometric mean peak moxifloxacin concentration (1733 ng/mL) was above 5 ms, thereby demonstrating assay sensitivity.

### Safety Results

A total of 45 treatment‐emergent AEs (TEAEs) were reported by 20 (64.5%) of the 31 participants who received ≥1 dose of the study drug (Table 2). Most (41) TEAEs reported were mild in severity. Moderate TEAEs were reported by three participants after they received givinostat 300 mg. One participant reported a headache, and one reported abdominal pain and pain in the jaw. Increased blood triglycerides (395.9 mg/dL) occurred in one participant. The most commonly reported TEAEs during this study were nausea (19.4%), diarrhea (12.9%), and abdominal discomfort (9.7%). Two participants reported clinically significant laboratory abnormalities that led to TEAEs. There were no clinically significant changes in vital signs and no TEAEs related to vital signs reported during the study. No deaths, serious or severe TEAEs, discontinuations caused by a TEAE, or SUSARs were reported during the study, and all TEAEs resolved by the end of the study.

**Table 2:** Summary of Adverse Events in the Safety Population

	Givinostat 100 mg, n = 29	Givinostat 300 mg, n = 31	Placebo, n = 31	Moxifloxacin, n = 31	Overall, n = 31
At least 1 TEAE, n (%)	5 (17.2%)	15 (48.4%)	6 (19.4%)	4 (12.9%)	20 (64.5%)
Number of TEAEs	6	25	8	6	45
Serious TEAEs	0	0	0	0	0
Severe TEAEs	0	0	0	0	0
Related TEAEs	6	20	6	4	36
Discontinued owing to TEAE, n (%)	0	0	0	0	0
Number of deaths	0	0	0	0	0
Most frequently reported TEAE,^a^ n (%); E					
Nausea	0	5 (16.1%); 5	0	2 (6.5%); 2	6 (19.4%); 7
Diarrhea	2 (6.9%); 2	1 (3.2%); 1	2 (6.5%); 2	0	4 (12.9%); 5
Abdominal discomfort	0	3 (9.7%); 3	0	0	3 (9.7%); 3
Headache	1 (3.4%); 1	4 (12.9%); 4	1 (3.2%); 1	0	4 (12.9%); 6

E, number of TEAEs; TEAE, treatment‐emergent adverse event.

a TEAEs reported by >1 participant who received the study drug.

## Discussion

The primary objective of the study was to evaluate the effect of givinostat at therapeutic (100 mg) and supratherapeutic (300 mg) doses on the QT/QTc interval, with a ΔΔQTcF exceeding 10 ms considered clinically relevant. The maximum observed mean ΔΔQTcF with the 100‐mg dose was 5.5 ms (90% CI, 1.99 to 9.01), suggesting that a clinically relevant QT prolongation (i.e., exceeding 10 ms) can be excluded with this dose. Additionally, the effect on HR with givinostat 100 mg was small and of no clinical concern. In previous studies evaluating a broader dose range, givinostat exposure was reported to increase proportionally with dose.^19^ In the present study, which evaluated only two dose levels (100 and 300 mg), exposure increased more than the corresponding increase in dose between these levels; however, formal dose proportionality could not be assessed in the present study, given the evaluation of only two dose levels.

The supratherapeutic dose of givinostat (300 mg) produced a clinically relevant effect on both HR and ΔΔQTcF, with a peak ΔΔQTcF of 13.6 ms. This peak was delayed relative to the maximum givinostat plasma concentration, indicating the presence of hysteresis. Following the givinostat 300‐mg dose, the mean ΔΔQTcF remained at or slightly >10 ms until 24 h postdose and thereafter declined. In the concentration‐QTc analysis, a linear model with a treatment effect‐specific intercept was fitted for plasma concentrations. The model did not adequately capture the data, as it appeared to overestimate the effect at higher concentration levels. Owing to the observed hysteresis, the linear concentration‐QTc model seemed to be mis‐specified, and an E_max_ model was subsequently used to predict the QTc effect at high givinostat concentration levels. The treatment effect‐specific intercept from the E_max_ model was small and not statistically significant, while the treatment effect‐specific intercept from the linear mixed‐effects model was large and statistically significant. An E_max_ model can therefore be used to better understand the predicted QTc effect at high concentration levels, which indicates that a ΔΔQTcF exceeding 10 ms can be excluded for the full range of plasma concentrations observed in this study and up to ∼745 ng/mL.

Although delayed metabolite exposure may contribute to the observed hysteresis,^27^ preclinical data indicate that givinostat metabolites do not directly inhibit human ether‐a‐go‐go (hERG) channels, consistent with a temporal PK–PD dissociation rather than a hERG‐mediated effect (data on file). In line with this observation, the two primary givinostat metabolites (ITF2375 and ITF2374) are approximately 1000 times less pharmacologically active than givinostat. The C_max_ for the metabolites occurred later than that of givinostat. In contrast, the highest mean plasma concentrations (C_max_) of givinostat were observed at 2 h postdose for both the 100‐ and the 300‐mg dose. The difference in elimination half‐life between givinostat 300 mg and givinostat 100 mg was approximately 3 h and was considered minimal. The urine excretion profile was similar for both givinostat doses, with slightly >1% of the administered dose recovered in urine.

Overall, the oral administration of therapeutic and supratherapeutic doses of givinostat was safe and well tolerated in healthy adults. The results of this study are consistent with a previous givinostat study in healthy participants in which givinostat was safe with no clinically significant changes in ECG parameters or QT prolongation.^19^ QTc has been consistently monitored in clinical trials involving patients with DMD, and no QTc‐related AEs have been reported with givinostat treatment. In a post hoc analysis of ECG parameters from the EPIDYS trial, no QTc prolongation was observed after givinostat treatment in patients with DMD^17^, ^28^ and the safety data in an ongoing open‐label, long‐term safety, tolerability, and efficacy study of givinostat in boys with DMD (NCT03373968) indicate that no patients had a QTcF >450 ms or an increase of >60 ms in QTcF intervals.^17^ A PPK analysis study including seven clinical trials suggested that givinostat dosing should be adjusted based on body weight.^21^ The PPK analysis also showed that PK profiles are similar between healthy participants and a clinical population, including patients with DMD and systemic‐onset juvenile idiopathic arthritis.^21^ The current dose recommendations for givinostat^2^ were informed by a PPK analysis aimed at identifying doses that result in an exposure of ≈570 ng × h/mL, which is 3.3‐fold lower than the exposure observed with the 300‐mg dose in this study (1893.6 ng × h/mL).^21^ Simulated steady‐state exposures were comparable across body weights and remained below 500 ng × h/mL,^29^ consistent with exposures observed with the 100‐mg dose in this study and well below those observed with the 300‐mg dose.

Several HDAC inhibitors, including vorinostat, romidepsin, and belinostat, have been approved by the FDA for the treatment of cancer, primarily cutaneous T‐cell lymphoma and peripheral T‐cell lymphoma.^30^ A range of SAEs have been reported with these drugs, particularly myelosuppression, diarrhea, and cardiotoxicity.^10^ A systematic review of 62 studies, including 3268 patients, found that QTc prolongation occurred in 4.4% of patients receiving HDAC inhibitor therapy for cancer.^11^ Of the FDA‐approved drugs for cancer therapy, this event occurred in 12% of patients receiving belinostat and 3% of patients receiving vorinostat and romidepsin.^11^ However, the totality of clinical data suggests that HDAC inhibitors do not pose significant safety issues as a class, and cardiotoxic effects are uncommon and manageable through dose adjustments.^31^ Combination drug treatment with HDAC inhibitors and other medications, including certain antiemetics, antibiotics, and antidepressants, may increase QTc prolongation risk owing to pharmacodynamic and PK drug interactions.^32^ Taken together, the findings indicate that monitoring of cardiac function is advisable before initiating HDAC inhibitor treatment and during treatment.

Givinostat was developed as a novel HDAC inhibitor with an improved cardiac safety profile. HDAC inhibitors have been shown to disrupt the function of hERG channels, potentially leading to QTc prolongation.^33^ However, givinostat is unlikely to directly affect hERG channels based on preclinical studies, as the maximal circulating concentrations in adults and pediatric patients at the highest evaluated doses were approximately 90‐ to 140‐fold lower than the IC_50_ for hERG inhibition (data on file). Accordingly, the QTc effects observed in this study at the supratherapeutic givinostat dose are not considered to reflect direct hERG channel inhibition. In vitro studies have shown that HDAC inhibitors that have increased specificity for class I HDACs might have an enhanced cardiac safety profile.^34^ Although givinostat is considered a pan‐inhibitor, it is highly specific for class I and class II HDACs, while other HDAC inhibitors, including vorinostat and belinostat, are less selective.^30^ In summary, this thorough QT study provides support that a therapeutic dose of givinostat is not associated with risk of QTc prolongation.

## Conclusion

Clinically relevant QTc prolongation was observed only with the supratherapeutic dose of givinostat, which is approximately five times the dose recommended for patients with DMD weighing 60 kg or more. The LS mean ΔΔQTcF reached a peak of 5.5 ms at 5 h postdosing with givinostat 100 mg. As such, givinostat at the therapeutic dose is not expected to pose a risk of QTc prolongation. Given that cardiovascular events have been observed in oncology clinical trials with different HDAC inhibitors,^11^ QTc assessment should be required to determine patient eligibility for clinical trials with givinostat or for in‐clinic treatment initiation.^2^

## Conflicts of Interest

Eugenio Mercuri has received payment or honoraria for lectures and symposia from PTC Therapeutics, Roche, and Sarepta and has participated on advisory boards for Dyne, Italfarmaco S.p.A., NS Pharma, Pfizer, PTC Therapeutics, Roche, Santhera, Sarepta, and WAVE Life Sciences. Barry Byrne has participated on advisory boards for Edgewise, North America Pompe Registry, and Pfizer Global DMD. Tracey Willis has received personal fees from Biogen, Genzyme, Newcastle University, Novartis, PTC Therapeutics, Sanofi, Santhera, and Sarepta. John Bourke has received data monitoring committee participation fees from Sarepta, travel support from Sarepta, and consulting and advisory board fees from Esperare Foundation, Pfizer, and Sarepta. Paolo Bettica and Sara Cazzaniga are employees of Italfarmaco S.p.A. Hongqi Xue is an employee of Clario. Borje Darpo works as a consultant for Clario and owns stocks and stock options in Clario.

## Funding

This study was funded by Italfarmaco S.p.A. Medical writing support was provided by Diane Kim, PhD, from Citrus Health Group, Inc. (Chicago, Illinois) and was funded by Italfarmaco S.p.A.

## Supporting information



Supporting Information

## Data Availability

The data that support the findings of this study are available on request from the corresponding author. The data are not publicly available due to privacy or ethical restrictions.
