# Supplementary material for: Thorough QT Study on the Effect of Therapeutic and Supratherapeutic Dosing of Givinostat in Healthy Volunteers
Source: Clin Pharmacol Drug Dev. 2026 Mar 22;15(3):e70047. doi: 10.1002/cpdd.70047 (PMC13006724; doi:10.1002/cpdd.70047)
Supplement: Supplementary file 1 — Supporting Information [file CPDD-15-0-s001.docx]

**Supplemental Material**

**Inclusion and Exclusion Criteria**

*Inclusion Criteria*

Participants were eligible for inclusion in the study if they met all the following inclusion criteria

1. Men or women, nonsmoker as defined as no use of tobacco or nicotine products within 3 months prior to screening, ≥18 and ≤55 years of age, with body mass index >18.5 and <30.0 kg/m^2^ and body weight ≥55 kg and ≤100 kg for women and body weight ≥60 kg and ≤100 kg for men.
2. Participants needed to be healthy as defined by the absence of clinically significant (CS) illness and major surgery within 4 weeks prior to dosing as well as the absence of CS history of neurologic, endocrine, cardiovascular, pulmonary, hematologic, immunologic, psychiatric, gastrointestinal, renal, hepatic, and metabolic disease. Participants vomiting within 24 hours predose were carefully evaluated for upcoming illness/disease. Inclusion predosing of the participant in the study was at the discretion of the investigator, depending on his/her clinical judgement.
3. Women had to be of nonchildbearing potential, which was defined as postmenopausal (absence of menses for 12 months prior to the first study drug administration, bilateral oophorectomy or hysterectomy with bilateral oophorectomy ≥6 months prior to the first study drug administration) or surgically sterile (hysterectomy or tubal ligation ≥6 months prior to drug administration).
4. Women of childbearing potential who were sexually active with a male partner must have been willing to use 1 of the following acceptable contraceptive methods throughout the study and for ≥90 days after the last study drug administration: simultaneous use of intrauterine contraceptive device, without hormone release system placed ≥4 weeks prior to study drug administration, and condom for the male partner or simultaneous use of diaphragm or cervical cap with intravaginally applied spermicide and condom for the male partner, started ≥21 days prior to study drug administration.
5. Male participants who were not vasectomized for ≥6 months prior to first study drug administration and who were sexually active with a female partner of childbearing potential (childbearing potential women were defined as those not postmenopausal or surgically sterile) must have been willing to use 1 of the following acceptable contraceptive methods from the first study drug administration until ≥90 days after the last study drug administration: simultaneous use of a male condom and hormonal contraceptives used for ≥4 weeks or intrauterine contraceptive device placed for ≥4 weeks for the female partner or simultaneous use of a male condom and a diaphragm or cervical cap with intravaginally applied spermicide for the female partner.
6. Male participants (including men who have had a vasectomy) with a pregnant partner must have agreed to use a condom from the first study drug administration until ≥90 days after the last study drug administration.
7. Male participants must have been willing to not donate sperm until 90 days following the last study drug administration.
8. Female participants must have been willing to not donate ovules until 90 days following the last study drug administration.
9. Participant’s written informed consent was obtained prior to any study-related procedure.
10. Participants were willing and capable to comply with the requirements of the study and were able to understand the study procedures and the risks involved.
11. Participants must have been willing to take out dentures and mouth piercings for study procedures.

*Exclusion Criteria*

Participants were excluded if any of the following exclusion criteria were met

1. Presence of any CS abnormality at physical examination, CS abnormal laboratory test results, or positive test for HIV, hepatitis B, or hepatitis C found during medical screening.
2. CS vital sign abnormalities (systolic blood pressure [BP] <90 or >140 mmHg, diastolic BP <60 or >90 mmHg, or HR <40 or >100 beats per minute) at screening. For eligibility purposes, 2 single measurements were considered, not the mean value.
3. Any of the following abnormalities on 12-lead electrocardiogram (ECG) at screening: PR interval ˃210 msec; QRS complex ˃120 msec; QTcF ˃450 msec; any abnormality of cardiac rhythm other than sinus arrhythmia; or abnormality of T-wave morphology that would impair the ability to measure the QT interval reliably. The averaged value of 3 ECGs, 5 minutes apart from each other, were used; evaluations were used for the evaluation of the QTc interval requested by this exclusion criteria.
4. Participants with history of sustained and nonsustained cardiac arrhythmias (ECG demonstrated), participants with a family history of sudden cardiac death, and participants with a history of additional risk factors for torsade de pointes, heart failure, hypokalaemia, or long QT syndrome.
5. Abnormal laboratory test values at screening or at baseline (day –1) of Period 1 of platelet count <125 x 10^9^/L or absolute neutrophil count <1.2 x 10^9^/L.
6. Participants who had cardiovascular condition such as, but not limited to, unstable ischemic heart disease; New York Heart Association class III/IV left ventricular failure; or acute ischemic heart disease in the past year prior to study screening, which may impact the safety of the participant or the evaluation of the result of the study according to the investigator's judgment. Cardiovascular conditions were discarded based on the results obtained on the ECG, medical examination, and routine lab test.
7. Positive urine drug screen, alcohol breath test, or urine cotinine test at screening or at baseline (day –1).
8. History of anaphylaxis reaction or CS drug hypersensitivity reaction (eg, angioedema, Stevens-Johnson syndrome, acute generalized exanthematous pustulosis, drug-induced hypersensitivity syndrome, drug-induced neutropenia).
9. History of allergic reactions to givinostat, histone deacetylase inhibitors, or other related drugs; moxifloxacin; other quinolones; or to any excipient in the formulation.
10. Positive pregnancy test at screening or at baseline (day –1).
11. Participants with a sorbitol intolerance or sorbitol malabsorption or had fructose intolerance.
12. Current or recent (within 3 months of study drug administration) CS gastrointestinal disease that could interfere with drug absorption.
13. Gastrointestinal surgery that interfered with physiologic absorption and motility (ie, gastric bypass, duodenectomy) or gastric bands.
14. History of significant alcohol abuse within 1 year prior to screening or regular use of alcohol within 6 months prior to the screening visit (>14 units of alcohol per week [1 unit = 150 mL of wine, 360 mL of beer, or 45 mL of 40% alcohol]).
15. History of significant drug abuse within 1 year prior to screening or use of soft drugs (such as marijuana) within 3 months prior to the screening visit or hard drugs (such as cocaine, phencyclidine, crack, opioid derivatives including heroin, and amphetamine derivatives) within 1 year prior to screening.
16. Use of givinostat for a medical condition or in the context of another clinical trial within a period of 30 days prior to the first dosing.
17. Participation in a clinical research study involving the administration of an investigational or marketed drug or device within 30 days prior to the first dosing, administration of a biological product in the context of a clinical research study within 90 days prior to the first dosing, or concomitant participation in an investigational study involving no drug or device administration.
18. Use of medications for the time frames specified below, with the exception of medications exempted by the investigator on a case-by-case basis because they were judged unlikely to affect the pharmacokinetic profile of the study drug or participant safety (eg, topical drug products without significant systemic absorption): prescription medications within 14 days prior to the first dosing; over-the-counter products (with the exception of the occasional use of acetaminophen [up to 2 g daily]) and natural health products (including herbal remedies, homeopathic and traditional medicines, probiotics, food supplements such as vitamins, minerals, amino acids, essential fatty acids, and protein supplements used in sports) within 7 days prior to the first dosing; depot injection or implant of any drug within 3 months prior to the first dosing; or any drugs known to induce or inhibit hepatic drug metabolism (including St. John’s wort) within 30 days prior to the first dosing.
19. Donation of plasma within 7 days prior to dosing. Donation or loss of blood (excluding volume drawn at screening) of 50 mL to 499 mL of blood within 30 days, or >499 mL within 56 days prior to the first dosing.
20. Participant that was breastfeeding.
21. Participant who was unable to be venipunctured and/or tolerate catheter venous access.
22. Participant who was unable or had difficulties swallowing tablets or suspension.
23. Any reason which, in the opinion of the investigator, would prevent the participant from participating in the study.
24. History or presence of other diseases, metabolic dysfunctions, physical examination findings, or any clinically relevant abnormal laboratory value at screening suggesting an unknown disease and requiring further clinical investigation or that may have impacted the safety of the participant or the evaluation of the result of the study according to the investigator's judgment.

**Description of the E_max_ Model**

ΔQTcF_ijk_ = E_0_ × TRT_j_ + r_i1_ + β_k_ × t_k_ + α × (B_QTc,i_ – B) + (E_max_ + ri_2_) × C_ijk_ / (EC_50_ + C_ijk_) + ɛ_ijk_

Based on the model, i is the i-th participant, j is the j-th treatment, k is the k-th time point, TRT_j_ is the j-th treatment effect (active=1 or placebo=0), t_k_ is the k-th time effect, B_QTc,i_ is the baseline QTcF for the i-th participant, B is the population mean baseline QTcF, C_ijk_ is the plasma concentration of ITF2357 at the k-th time point for treatment j for participant i. The parameter E_0_, is the effect when the drug concentration is zero, E_max_ is the maximum effect attributable to the drug and EC_50_ is the concentration, which produces half of E_max_. β_k_ and α are the corresponding coefficients for time effect tk and centered baseline QTcF, respectively. r_i1_ and r_i2_ are the participant-specific random effects for the intercept and E_max_, respectively, and assumed to be iid (ie, independent and identically distributed) normal N([0,0],[s2r1,0,s2r2]), and the error term ɛ_ij_ was assumed to be iid normal N(0, s2e) and independent of (ri1, ri2).

**Figures**

**Figure S1.** Participant disposition

^a^Enroled participants include those who were deemed eligible and accepted to participate in the trial after having signed the approved final version of the study informed consent form, as well as those identified as standby who may replace participants who withdraw from the study before dosing.

^b^n=2 not dosed with givinostat 100 mg owing to invalid laboratory results.

PK, pharmacokinetic; QTc, corrected QT.

**Figure S2.** Mean (SD) givinostat plasma concentrations over time (PK/QTc population)

******

If mean (SD) was below 0, 0 was substituted as concentrations <0 are not biologically plausible.

PK, pharmacokinetic; QTc, corrected QT; ST, supratherapeutic; T, therapeutic.

**Figure S3.** Model-predicted ΔΔQTc and estimated placebo-corrected ΔQTcF across deciles of givinostat plasma concentrations for givinostat E_max_ model (PK/QTc population)

***
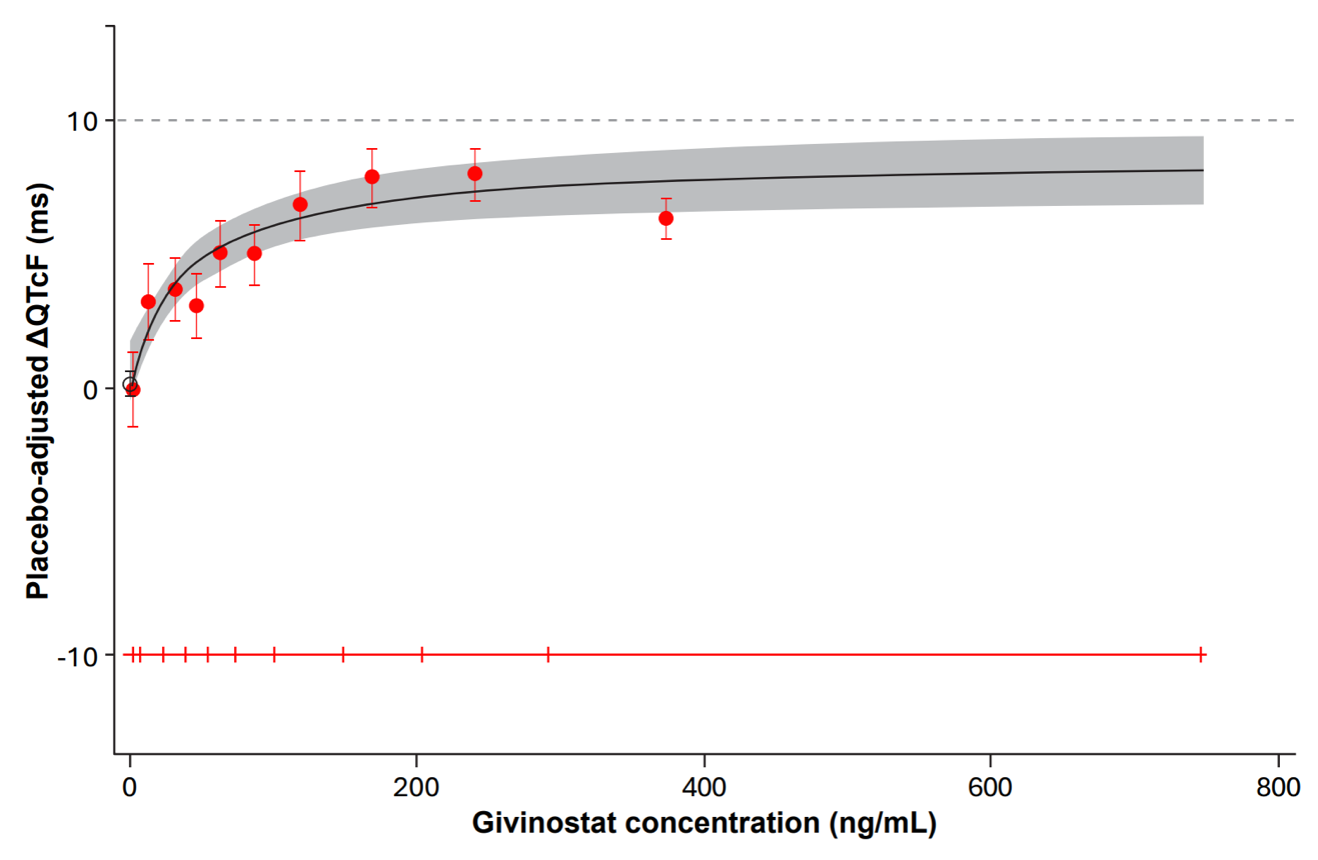
***

The red filled circles with vertical bars denote the mean placebo-corrected ΔQTc (ΔΔQTc) with 90% CI displayed at the median plasma concentration within each decile for givinostat. The solid black line with gray shaded area denotes the model-predicted mean placebo-adjusted ΔQTc with 90% CI. The horizontal red line with notches shows the range of concentrations divided into deciles for givinostat. The distance between each decile represents the point at which 10% of the data is present; the first notch to second notch denotes the first 10% of the data, the second notch to third notch denotes the 10-20% of the data and so on.

PK, pharmacokinetic; QTc, corrected QT.

**Tables**

**Table S1.** Summary of plasma and urine pharmacokinetic parameters of givinostat 100 mg and 300 mg and metabolites and moxifloxacin (PK population)

|  | Givinostat 100 mg  n=29 | | | Givinostat 300 mg n=29 | | | Moxifloxacin  n=29 |
| --- | --- | --- | --- | --- | --- | --- | --- |
|  | **Givinostat**  **n=29** | **ITF2374**  **n=12** | **ITF2375**  **n=12** | **Givinostat**  **n=29** | **ITF2374**  **n=12** | **ITF2375**  **n=12** |  |
| Plasma PK mean (SD) | | | | | | | |
| AUC_0-t_ (h*ng/mL) | 610.6 (137.8) | 435.7 (278.1) | 3134.9 (1720.5) | 2361.5 (484.8) | 1486.5 (886.7) | 11173.5 (5888.0) | 27423.4  (4434.5) |
| AUC_0-12_ (h*ng/mL) | 480.7 (114.1) | 178.1 (81.6) | 1680.8 (725.8) | 1893.6 (423.3) | 607.2 (258.7) | 6018.0 (2430.1) | 13625.1  (2733.5) |
| AUC_0-inf_ (h*ng/mL) | 626.6 (139.5) | 475.8 (318.3) | 3247.0 (1806.5) | 2383.3 (484.7) | 1555.7 (986.4) | 11373.9 (6045.3) | 28327.2  (4616.5) |
| C_max_ (ng/mL) | 102.8 (30.6) | 24.4 (11.0) | 258.6 (111.2) | 409.7 (133.7) | 81.6 (32.9) | 820.8 (273.5) | 1789.5 (498.1) |
| T_max_ (h), median (min, max) | 2.1 (1.1, 3.4) | 5.1 (5.1, 6.3) | 3.6 (2.2, 4.1) | 2.2 (1.2, 5.1) | 5.6 (5.1, 7.1) | 4.1 (2.6, 5.1) | 2.3 (0.6, 4.1) |
| T_½ el_ (h) | 8.0 (2.2) | 13.1 (4.6) | 12.6 (3.3) | 11.1 (2.4) | 13.9 (4.6) | 10.7 (2.0) | 14.5 (2.8) |
| K_el_ (h) | 0.09 (0.02) | 0.06 (0.01) | 0.06 (0.02) | 0.07 (0.01) | 0.05 (0.02) | 0.07 (0.01) | 0.05 (0.01) |
| CL/F (L/h) | 167.1 (36.4) | 296.9 (172.7) | 43.1 (27.8) | 131.1 (27.5) | 251.8 (124.7) | 36.7 (24.0) | 14.5 (2.4) |
| V_d_/F (L) | 1897.0 (588.2) | 5094.9 (2676.4) | 795.3 (636.3) | 2125.5 (764.3) | 5027.5 (3106.9) | 576.4 (436.7) | 303.0 (72.2) |
| MRAUC | - | 0.76 (0.5) | 5.30 (2.8) | - | 0.68 (0.5) | 4.99 (2.8) | - |
| MRC_max_ | - | 0.25 (0.1) | 2.65 (0.8) | - | 0.22 (0.1) | 2.16 (0.7) | - |
| Urine PK mean (SD) | | | | | | | |
| Ae_0-t_ (ug) | 1304.8 (444.6) | 1526.6 (820.3) | 330.0 (244.2) | 4284.1 (1031.5) | 4634.6 (2469.8) | 1051.8 (702.8) | - |
| R_max_ (ug/h) | 304.9 (327.3) | 155.7 (166.3) | 51.6 (46.1) | 1224.7 (922.9) | 536.1 (361.7) | 182.3 (172.6) | - |
| T_Rmax_ (h) | 1.9 (1.0) | 4.9 (4.1) | 2.4 (2.5) | 2.1 (2.4) | 4.2 (3.9) | 2.1 (2.4) | - |
| Clr (L/h) | 2127.6 (402.6) | 3856.3 (979.4) | 103.4 (27.9) | 1859.3 (360.8) | 3403.5 (1004.8) | 96.0 (29.3) | - |

ITF2374 and ITF2375 are metabolites of givinostat.

CL/F is calculated as dose/AUC_0-inf_.

V_d_/F, calculated as dose/K_el_ × AUC_0-inf_.

Ae_0-t_, cumulative urinary excretion from time zero to time t; AUC_0-t_, area under the concentration-time curve from time zero to time of the last nonzero concentration; AUC_0-12_, area under the concentration-time curve from time zero to 12 hours; AUC_0-inf_, area under the concentration-time curve from time zero to infinity (extrapolated); CL/F, apparent total body clearance; Clr, renal clearance; C_max_, maximum observed plasma concentration; K_el_, elimination rate constant; MRAUC, metabolite to parent ratio for AUC; MRC_max_, metabolite to parent ratio for C_max_; PK, pharmacokinetics; R_max_, maximum rate of urinary excretion; t_1/2el_, elimination half-life, calculated as ln(2)/K_el_; T_max_, time of observed C_max_; T_Rmax_, time R_max_, calculated as the midpoint of the collection interval during which R_max_ occurred; V_d_/F, apparent volume of distribution.

**Table S2.** Concentration-QTc analysis of givinostat E_max_ model and associated ΔQTc prolongation (PK/QTc set)

| **Parameter** | **Estimate** | **SE** | ***df*** | ***t-*Value** | ***P*Value** | **90% CI** |
| --- | --- | --- | --- | --- | --- | --- |
| Treatment-effect Intercept (ms) | 0.08 | 0.96 | 29.0 | 0.08 | 0.9360 | -1.55, 1.71 |
| E_max_ (ms) | 8.45 | 1.15 | 29.0 | 7.38 | < 0.0001 | 6.50, 10.40 |
| ED_50_ (ng/mL) | 40.96 | 15.60 | 29.0 | 2.62 | 0.0137 | 14.44, 67.47 |
| Centered baseline Effect (ms) | -0.18 | 0.03 | 29.0 | -6.18 | < 0.0001 | -0.23, -0.13 |
| Day 1: 0.5 h postdose effect (ms) | -4.38 | 0.81 | 29.0 | -5.40 | < 0.0001 | -5.76, -3.00 |
| Day 1: 1 h postdose effect (ms) | -4.07 | 0.81 | 29.0 | -5.00 | < 0.0001 | -5.45, -2.69 |
| Day 1: 1.5 h postdose effect (ms) | -2.99 | 0.82 | 29.0 | -3.66 | 0.0010 | -4.38, -1.60 |
| Day 1: 2 h postdose effect (ms) | -2.68 | 0.82 | 29.0 | -3.28 | 0.0027 | -4.07, -1.29 |
| Day 1: 2.5 h postdose effect (ms) | -1.28 | 0.82 | 29.0 | -1.57 | 0.1265 | -2.67, 0.10 |
| Day 1: 3 h postdose effect (ms) | -0.30 | 0.81 | 29.0 | -0.37 | 0.7161 | -1.68, 1.08 |
| Day 1: 3.5 h postdose effect (ms) | 2.02 | 0.81 | 29.0 | 2.49 | 0.0188 | 0.64, 3.40 |
| Day 1: 4 h postdose effect (ms) | 3.39 | 0.81 | 29.0 | 4.19 | 0.0002 | 2.02, 4.77 |
| Day 1: 5 h postdose effect (ms) | 1.49 | 0.81 | 29.0 | 1.84 | 0.0761 | 0.11, 2.87 |
| Day 1: 6 h postdose effect (ms) | -1.40 | 0.81 | 29.0 | -1.72 | 0.0955 | -2.77, -0.02 |
| Day 1: 7 h postdose effect (ms) | -4.09 | 0.81 | 29.0 | -5.04 | < 0.0001 | -5.47, -2.71 |
| Day 1: 8 h postdose effect (ms) | -3.80 | 0.81 | 29.0 | -4.68 | < 0.0001 | -5.18, -2.42 |
| Day 1: 12 h postdose effect (ms) | 3.48 | 0.82 | 29.0 | 4.25 | 0.0002 | 2.09, 4.87 |
| Day 1: 24 h postdose effect (ms) | 0.17 | 0.88 | 29.0 | 0.20 | 0.8464 | -1.33, 1.67 |
| Day 1: 36 h postdose effect (ms) | -1.10 | 0.93 | 29.0 | -1.18 | 0.2462 | -2.69, 0.48 |

Based on a non-linear mixed model with ΔQTc as the dependent variable, (givinostat × E_max_) / (ED_50_ + givinostat) as a continuous covariate, centered baseline QTc as an additional covariate, treatment and time as categorical factors, and a random intercept and slope of E_max_ per participant.
ED_50_, median effective dose; E_max_, maximum effect; PK, pharmacokinetic; QTc, corrected QT.
